# Functional distinctness in the exoproteomes of marine *S* *ynechococcus*

**DOI:** 10.1111/1462-2920.12822

**Published:** 2015-04-15

**Authors:** Joseph A. Christie‐Oleza, Jean Armengaud, Philippe Guerin, David J. Scanlan

**Affiliations:** ^1^ School of Life SciencesUniversity of WarwickCoventryCV4 7ALUK; ^2^ CEADSVIBiTec‐SSPILi2DLaboratory ‘Technological Innovations for Detection and Diagnostic’Bagnols‐sur‐CèzeF‐30207France

## Abstract

The exported protein fraction of an organism may reflect its life strategy and, ultimately, the way it is perceived by the outside world. Bioinformatic prediction of the exported pan‐proteome of *P* *rochlorococcus* and *S* *ynechococcus* lineages demonstrated that (i) this fraction of the encoded proteome had a much higher incidence of lineage‐specific proteins than the cytosolic fraction (57% and 73% homologue incidence respectively) and (ii) exported proteins are largely uncharacterized to date (54%) compared with proteins from the cytosolic fraction (35%). This suggests that the genomic and functional diversity of these organisms lies largely in the diverse pool of novel functions these organisms export to/through their membranes playing a key role in community diversification, e.g. for niche partitioning or evading predation. Experimental exoproteome analysis of marine *S* *ynechococcus* showed transport systems for inorganic nutrients, an interesting array of strain‐specific exoproteins involved in mutualistic or hostile interactions (i.e. hemolysins, pilins, adhesins), and exoenzymes with a potential mixotrophic goal (i.e. exoproteases and chitinases). We also show how these organisms can remodel their exoproteome, i.e. by increasing the repertoire of interaction proteins when grown in the presence of a heterotroph or decrease exposure to prey when grown in the dark. Finally, our data indicate that heterotrophic bacteria can feed on the exoproteome of *S* *ynechococcus*.

## Introduction

The exoproteome is the protein fraction found in the extracellular proximity of one or several organisms. It comprises actively exported proteins and those that are released by cell lysis or leakage. For example, the exoproteomes of pure cultures are those proteins that can be detected after purification from the culture medium once cells have been removed (Armengaud *et al*., 2012). These proteins, present in hostile extracellular conditions, need long half‐lives in order to accumulate and, hence, be detected. Protein translocation through the cytoplasmic membrane is believed to be mainly driven by the Sec and Tat pathways (see e.g. Barnett *et al*., 2011 for a review of the cyanobacterial Tat system), while up to eight different translocation systems carry proteins through the cytoplasmic and outer membranes of gram‐negative bacteria (Saier, 2006), three of which secrete proteins directly though the gram‐negative bilayer (type I, III and IV). To date, extensive exoproteomes of environmental bacteria are poorly described, particularly for marine microorganisms, with only two single case studies in the γ‐proteobacterium *Pseudoalteromonas tunicata* and the α‐proteobacterium *Ruegeria pomeroyi* (Evans *et al*., 2007; Christie‐Oleza and Armengaud, 2010), and a comprehensive analysis of marine *Roseobacters* (Christie‐Oleza *et al*., 2012). This latter analysis of the exoproteome among members of the *Roseobacter* clade showed different adaptive life strategies among the studied strains inferred by their exported proteins (Christie‐Oleza *et al*., 2012).

Picocyanobacteria comprise a key component of the marine picoplankton being major contributors to primary production and underpinning the marine food web due to their large abundance (Partensky *et al*., 1999; Jardillier *et al*., 2010). Two genera, *Prochlorococcus* and *Synechococcus*, numerically dominate marine waters, occupying complementary although overlapping oceanic regimes and with both genera exhibiting extensive genomic diversity (see Partensky *et al*., 1999; Kettler *et al*., 2007; Scanlan *et al*., 2009; Kashtan *et al*., 2014). The existence of hypervariable genomic regions (or islands) (Palenik *et al*., 2003; Coleman *et al*., 2006; Dufresne *et al*., 2008) has been linked with the importance of these regions in environmental adaptation (both biotic and abiotic) in both genera (Avrani *et al*., 2011; Stuart *et al*., 2013). Furthermore, closely related coexisting *Prochlorococcus* subpopulations according to their intergenic transcribed spacer showed a much higher divergence in terms of their genomic‐encoded auxilliary functions as a matter of avoiding competition and niche partitioning (Kashtan *et al*., 2014). Hence, the existing diversity of functions encoded by this group of photosynthetic organisms is somewhat far from being understood.

While numerous transcriptomic experiments have been performed on marine picocyanobacteria, studies using high‐throughput proteomics are more limited, e.g. quantitative proteomics of *Prochlorococcus* have been performed during light adaptation (Pandhal *et al*., 2007), a light‐dark cycle (Waldbauer *et al*., 2012), nitrogen starvation conditions (McDonagh *et al*., 2012) or from extracellular vesicles (Biller *et al*., 2014), while temperature shifts (Mackey *et al*., 2013) and nutrient depletion proteomes (Cox and Saito, 2013) are available for *Synechococcus*. However, very little is known with respect to exported proteins of marine picocyanobacteria with a few exceptions, e.g. the swimming and grazing defence proteins SwmA and SwmB in *Synechococcus* sp. WH8102 (McCarren and Brahamsha, 2007; Strom *et al*., 2012) and the PhoX alkaline phosphatase (Kathuria and Martiny, 2011). Here, we present the first exported pan‐proteome analysis of marine picocyanobacteria. Our initial bioinformatic prediction of the theoretical exoproteomes for a number of *Synechococcus* and *Prochlorococcus* strains clearly highlights the exported fraction as the major reservoir of proteins of unknown function and shows largest variability between strains. These observations suggest that the major discriminating parameter between strains lies within their exoproteome. Subsequent experimental analysis of the exoproteomes of eight *Synechococcus* strains revealed not only the expected importance of nutrient transport systems (e.g. those for phosphorus and iron) to these organisms, but also demonstrated expression of a wide range of poorly conserved exoenzymes with community interacting and mixotrophic implications. Both the need for novelty in the adaptation and survival of each strain in its specific environment and the weaker evolutionary constraints on proteins outside of cells may explain this pan‐exoproteome diversity.

## Results

### Pan‐genome analysis of the theoretical exoproteome of marine picocyanobacteria

**Figure 1:**
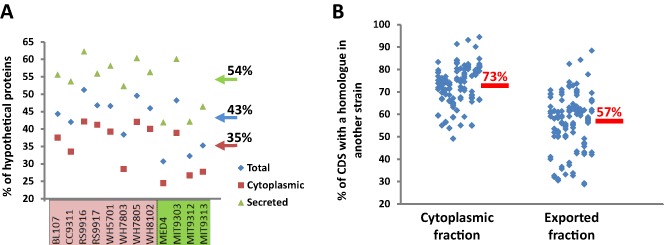
Theoretical exoproteomes of twelve marine picocyanobacterial strains. (A) Percentage of CDS annotated as hypothetical proteins in the cellular fractions (cytoplasmic and exported) of eight *S* *ynechococcus* strains (pink) and four *P* *rochlorococcus* strains (green). (B) Percentage of CDS from each strain and fraction (cytoplasmic and exported fractions) that found a homologous protein in each of the other eleven strains analysed in this study. Data were extracted from Table S2.

The exported proteins encoded by eight *Synechococcus* strains, encompassing members of several clades, four *Prochlorococcus* strains (encompassing two high light‐ and two low light‐adapted) and *Candidatus* Pelagibacter ubique (SAR11 in the text) were predicted using the prediction tools SignalP, SecretomeP, LipoP and PSORTb (Table S1). Table 1 reports the number of predicted exported proteins and their ratio compared with the whole theoretical proteome for each strain. These analyses showed that almost 40% of the total products of coding DNA sequences (CDS) of these organisms were predicted to be exported to the membrane or extracellular milieu. Figure 1A shows the ratio of uncharacterized proteins for the 12 picocyanobacteria. Remarkably, the proportion of proteins annotated as ‘hypothetical protein’ or with unknown function within the theoretically exported fraction (54 ± 7%) exceeded the overall theoretical proteome average (43 ± 7%) and was much higher than within the cytoplasmic fraction (35 ± 7%) (Fig. 1A).

**Table 1:** Theoretical exoproteomes

		Total CDS	Cytoplasmic fraction		Exported fraction	
			CDS	%	CDS	%
*Synechococcus*	BL107	2507	1563	62	941	38
	CC9311	2892	1676	58	1216	42
	RS9916	2961	1620	55	1341	45
	RS9917	2770	1717	62	1053	38
	WH5701	3346	2044	61	1302	39
	WH7803	2533	1482	59	1051	41
	WH7805	2883	1702	59	1181	41
	WH8102	2519	1604	64	915	36
*Prochlorococcus*	MED4	1717	1104	64	613	36
	MIT9303	2997	1676	56	1321	44
	MIT9312	1810	1160	64	650	36
	MIT9313	2269	1352	60	917	40
*Ca*. P. ubique	SAR11	1354	800	59	554	41
Average				60 ± 3		40 ± 3

A homology table where the CDS of each marine picocyanobacterium was searched against all other 11 strains used in this study (considering only BLASTp hits with an E‐value < 10^−20^) was constructed (Table S2). Figure 1B depicts for both fractions the global similarity level of their components. A considerably lower number of predicted exported CDS found a homologue in another strain (57% on average) when compared with the cytoplasmic fraction (73% on average), which highlights the ‘distinctness’ the exported protein fraction confers to individual strains (Fig. 1B). Even closely related *Synechococcus* strains such as WH7803 and WH7805 or high‐light *Prochlorococcus* MIT9312 and MED4 showed a higher proportion of homologous hits in the cytoplasmic fraction (91% and 95% respectively) as opposed to the exported protein fraction (82% and 88%).

Table 2 shows the functional grouping of proteins in eight clusters for the 12 picocyanobacteria. Proteins included in each cluster can be found in Table S2 and are further described in the Appendix S1.

**Table 2:** Functional grouping of picocyanobacterial theoretical exoproteomes

	*Synechococcus*								*Prochlorococcus*				%
	BL107	CC9311	RS9916	RS9917	WH5701	WH7803	WH7805	WH8102	MED4	MIT9303	MIT9312	MIT9313	
Transport systems	99	139	121	152	165	152	143	120	67	120	75	116	11.8
Cell wall structure/biogenesis	39	42	40	35	49	45	41	40	28	39	28	42	3.9
Photosynthesis/energy production	101	109	100	95	100	101	99	100	82	86	81	88	9.6
Coenzyme metabolism	20	17	18	17	23	16	19	16	17	17	14	18	1.8
Oxidative stress	15	16	15	14	18	16	16	13	12	14	11	15	1.4
Interaction and environment sensing	18	31	38	29	20	33	25	22	6	27	9	24	2.1
Others	140	230	211	171	206	159	162	115	160	259	167	217	18.3
Hypothetical/unknown	509	632	798	540	721	529	676	489	241	759	265	397	51.2
Total exported	941	1216	1341	1053	1302	1051	1181	915	613	1321	650	917	–

### Comparative experimental exoproteomes of eight *S* *ynechococcus* strains

**Figure 2:**
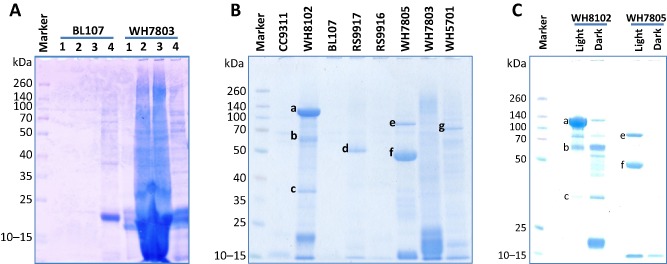
Exoproteomes of different marine *S* *ynechococcus* strains. Concentrated exoproteomes equivalent to 40 ml of culture were resolved by 10% Tris‐Bis NuPAGE gel (Invitrogen) and stained with coomassie G‐250 (SimplyBlue SafeStain, Invitrogen). Spectra Multicolor Broad Range Protein Ladder (Fermentas) was used as a marker. (A) Process for optimizing exoproteome preparation. The exoproteomes from *S* *ynechococcus* spp. BL107 and WH7803 were prepared from cultures: lane 1, washed cells in fresh ASW (10^7^ cells ml^−1^) and incubated for 2 days; lane 2, routinely subbed cultures (15% volume from an older culture in fresh ASW medium, resulting in 10^7^ cells ml^−1^) incubated for 2 days; lane 3, washed cells in fresh ASW (10^7^ cells ml^−1^) and incubated for 14 days; lane 4, protein extract of the cellular fraction from the corresponding *S* *ynechococcus* strain. (B) Exoproteomes from each one of the eight *S* *ynechococcus* strains analysed in this study by shotgun proteomics. Preparation of the exoproteomes was as indicated in the section *E* *xperimental procedures*. Letters indicate those resolved protein bands that were further identified using chymotrypsin digestion prior to LC‐MS‐MS: (a) SwmA NP_896180.1; (b) SwmA NP_896180.1 and SwmB NP_897046.1; (c) substrate binding protein of a phosphate ABC transporter NP_897111.1; (d) chitinase ZP_01081204.1; (e) type I secretion protein EAR18050.1; (f) haemolysin EAR19380.1 and chitinase EAR19694.1; (g) alkaline phosphatase EAQ75607.1. (C) Exoproteomes from light/dark incubated cultures of *S* *ynechococcus* sp. WH8102 and WH7805. Bands a, b, c, e and f are as those highlighted in panel B.

Figure 2 shows the SDS‐PAGE‐resolved exoproteomes of the eight *Synechococcus* strains. We observed that *Synechococcus* cultures generally accumulated proteins in the milieu in stationary phase possibly as a consequence of debris accumulation, especially in axenic cultures (Fig. 2A). Hence, cells were washed prior to transfer to avoid the carryover of proteins. Exoproteomes were subsequently prepared from culture supernatants (see *Experimental procedures*) following an increase in cell numbers from 3 × 10^7^ to 1 × 10^8^ cells ml^−1^ (see Fig. 2B where the equivalent of 40 ml of concentrated exoproteome from each strain was analysed by SDS‐PAGE). Shotgun nanoLC‐MS/MS proteomic analysis resulted in a total of 25 920 tryptic peptides being detected. Table 3 shows the number of non‐redundant peptides and proteins detected for each strain, as well as their functional categorization. We evaluated the fraction of proteins in the exoproteome that potentially originated from cell lysis by following the presence of ribosomal proteins. In all non‐axenic strains these proteins were not detected, while only a very low fraction was detected in the axenic strains (e.g. 1.1% in *Synechococcus* sp. WH8102; Table 3). For comparison, analysis of intracellular proteomes obtained from *Synechococcus* sp. WH8102 using the same culture conditions showed a much greater abundance of ribosomal proteins, comprising 8.8% of the proteome (data not shown) suggesting that even in the worst‐case scenario, < 13% of the exoproteomes could result from cell lysis under the conditions tested here. The non‐axenic *Synechococcus* strains produced a lower number of detected polypeptides as was evident from the SDS‐PAGE analysis (Fig. 2). *Synechococcus* sp. BL107 was the strain with the smallest number of polypeptides detected (11) despite re‐running samples through the mass spectrometer with four times more material. *Synechococcus* sp. BL107, unlike all other strains, showed almost no accumulation of proteins in the milieu (Fig. 2A). In contrast, a considerably higher number of polypeptides were detected in the axenic strains (WH5701, WH7803, WH7805 and WH8102), e.g. 247 polypeptides in *Synechococcus* sp. WH5701. Interestingly, 82% of the non‐axenic exoproteomes were predicted exported polypeptides whereas this was only 53% in the case of axenic strains. It is possible that the turnover of cytoplasmic proteins is higher in the extracellular milieu because these proteins may be potential targets for the exoprotease activity of heterotrophic bacteria as we discuss below. The detailed results of comparative exoproteomics between the eight strains are reported in Table S4 and commented on hereafter.

**Table 3:** Proteins detected by shotgun proteomics in the exoproteomes of eight marine *S* *ynechococcus* strains and abundance of these proteins across different functional groups

	Non‐axenic cultures				Axenic cultures			
	BL107	CC9311	RS9916	RS9917	WH5701	WH7803	WH7805	WH8102
Peptides (LC‐MS/MS runs)	340 (4)	1180 (2)	851 (2)	2325 (2)	7422 (2)	5420 (2)	2352 (2)	6030 (3)
Non‐redundant peptides	74	371	295	531	2071	1414	656	1231
Validated proteins (≥ 2 peptides)	11	60	42	67	247	202	84	146
**Non‐exported**	**3.2%**	**38.7%**	**15.8%**	**14.1%**	**57.5%**	**44.6%**	**34.0%**	**52.2%**
Ribosomal proteins	0.0%	0.0%	0.0%	0.0%	1.2%	0.6%	0.0%	1.1%
**Exported**	**96.8%**	**61.3%**	**84.2%**	**85.9%**	**42.5%**	**55.4%**	**66.0%**	**47.8%**
Transport systems	10.4%	5.0%	13.9%	9.2%	5.2%	0.5%	10.0%	6.5%
Cell wall structure/biogenesis	0.0%	0.4%	0.0%	2.2%	0.8%	0.5%	0.2%	0.5%
Photosynthesis/energy production	56.8%	17.8%	36.0%	7.8%	11.2%	18.0%	5.7%	19.9%
Oxidative stress	0.0%	1.3%	3.0%	4.1%	3.4%	3.9%	6.7%	5.2%
Interaction and environment sensing	16.5%	8.7%	7.7%	11.0%	2.3%	20.1%	8.6%	3.0%
Others	0.0%	14.2%	2.7%	11.8%	7.6%	4.2%	8.6%	4.7%
Hypothetical/unknown	13.1%	14.0%	20.9%	39.8%	12.1%	8.1%	26.0%	8.1%

#### Photosynthesis/energy production

Despite being proteins targeting the thylakoid membrane, this functional group of proteins is the most abundantly detected among the exoproteomes of *Synechococcus* (Table 3). This is principally due to the accumulation of their light harvesting pigments and associated phycobilisomes in the milieu (Table S4). For example, the four C‐phycoerythrin polypeptides (alpha/beta chains of class I/II C‐phycoerythrin, between 18–24 kDa in size; WH7805 only encodes class I) represent on average 26% of the exoproteome in the six strains in which they are encoded. Given the low abundance of ribosomal proteins (i.e. cytoplasmic non‐exported proteins) in our exoproteomes, suggesting low cell lysis, it is difficult to explain the presence of phycobilisome components in this fraction. The protein composition and dynamics in the plasma and thylakoid membrane systems remains a major challenge in cyanobacterial cell biology (Schneider, 2014) and, hence, it is possible these proteins are a result of mis‐targeting to the plasma membrane and beyond or if this is a consequence of thylakoid membrane blebbing and/or the production of extracellular vesicles (Biller *et al*., 2014).

#### Transport systems

Proteins involved in transporting substrates across the cell membrane were commonly found in the exoproteomes of *Synechococcus*, mainly ABC transporters and porins. ABC transporters specialized in transporting essential inorganic nutrients such as iron, phosphorus and, to a lesser extent, nitrogen were most abundant. The substrate binding protein of the iron ABC transporter was, on average, the sixth most abundant detected protein in our study mainly as it represented 8.7% and 4.6% of the exoproteome of strains RS9916 and RS9917 respectively (Table S4). The substrate binding protein of the phosphate ABC transporter was the seventh most abundant protein (Table S4) despite it being only abundantly detected in the axenic strains and the only transporter‐like protein detected in *Synechococcus* sp. WH7803. Interestingly, different isoforms of the phosphate binding protein were detected in strains WH5701, WH7805 and WH8102, suggesting that they may have subtly different functional roles, e.g. different binding affinities for phosphate. A potential component of a phosphonate ABC transport protein was detected (Table S4) in strains RS9917, WH5701 and WH7805 (the corresponding gene is conserved across picocyanobacterial genomes, see Scanlan *et al*., 2009) despite the fact that C‐P lyase, the enzyme required to cleave the recalcitrant C‐P bond of these compounds, has not been identified in any *Synechococcus* genome. Transporters for nitrogen were less abundant possibly because of the high N : P ratio (50:1) in ASW medium repressing their expression. Curiously, a potential urea ABC transporter protein was the most abundantly detected nitrogen transporter despite the fact that nitrate was the sole N source in ASW medium.

#### Oxidative stress

Proteins involved in oxidative stress were detected in the different exoproteomic fractions (Table 3). Superoxide dismutase (SOD), involved in detoxifying oxygen radicals, was detected in all strains except for BL107. Interestingly, all strains encoding the iron‐containing SOD produced this isoform (cluster 15 in Table S4). Those strains that lack the iron isoform of SOD, CC9311 and WH8102 produced the Cu/Zn and Ni isoforms respectively (cluster 106 and 99 in Table S4). Between one and three different thioredoxin peroxidase isoforms, with a function in cleaving hydrogen peroxide, were detected in all strains except for strains BL107 and CC9311 (clusters 21, 31, 232 and 287, Table S4). In *Synechococcus* sp. WH8102 however, we also detected a rubrerythrin‐like protein (comprising 2.9% of the exoproteome of this strain) playing a potential role in reducing hydrogen peroxide (Sztukowska *et al*., 2002).

#### Interaction and environment sensing

**Figure 3:**
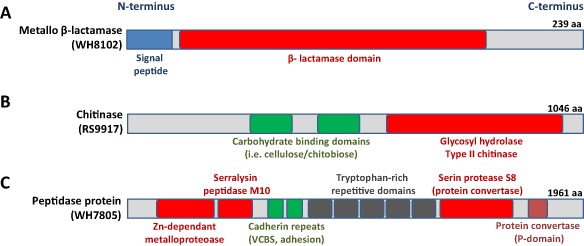
Domain structure of relevant exoproteins detected in marine *S* *ynechococcus* strains. (A) Highly conserved metallo β‐lactamase‐like protein abundantly detected in the exoproteomes of seven of the eight *S* *ynechococcus* strains analysed (NP_898405.1). (B) Abundantly detected chitinase‐like exoenzyme found in *S* *ynechococcus* spp. WH7803, WH7805, RS9916, RS9917, all with a similar domain structure (ZP_01081204.1). (C) Large protease‐like protein abundantly detected and unique to *Synechococcus* sp. WH7805 (EAR17767.1).

This group of proteins shows high strain‐specificity as detected proteins are generally unique to a particular strain or with only a few homologues. However, two of these exoproteins that do show a high degree of conservation are: (i) an abundant metallo‐β‐lactamase (with 1.3% average detection excluding BL107, cluster 16 in Table S4) with high identity across currently sequenced picocyanobacterial genomes (Fig. 3A) and (ii) the third most abundant polypeptide detected in our exoproteome survey (cluster 3, Table S4), annotated as a potential protein phosphatase 2C, that may play a key role in signal transduction (Fuchs *et al*., 2013). Interestingly, this protein phosphatase 2C is highly conserved and encoded by all eight *Synechococcus* strains.

Surprisingly, we abundantly detected a potential endo‐1–4‐β‐glycosyl hydrolase (a type II chitinase, cluster 12 in Table S4) in all those *Synechococcus* strains that encode this exoenzyme (i.e. 8.5% in RS9917; 2.0% in WH7805; 1.4% in RS9916; and 0.1% in WH7803) despite no substrate (chitin or similar) being added to the growth medium. All encoded chitinases display a similar structure, with carbohydrate‐binding domains at the N‐terminal half of the protein and a type II chitinase domain at the C‐terminus (Fig. 3B). These exoproteins have no signal peptide and are predicted to use a non‐classical mechanism for secretion. Interestingly, the gene encoding the *Synechococcus* sp. WH7803 chitinase lies immediately upstream of another annotated chitinase that was detected in similar abundance in the exoproteome of this strain (YP_001225792.1). Complete degradation of chitin requires β‐N‐acetylglucosaminidase (Gooday, 1990), although this enzyme was not detected in the experimental exoproteomes. β‐N‐acetylglucosaminidase is encoded in all of the picocyanobacterial genomes analysed in this study (except for the two HL *Prochlorococcus*), but is predicted to be a non‐exported protein, in accordance with its absence in the proteomics data.

The exoproteome of *Synechococcus* sp. WH7803 contained an extremely abundant pili‐like structure composed of proteins YP_001225518.1 and YP_001225519.1 representing 17.9% and 1.3% of the exoproteome respectively. No pili‐like proteins were detected in other strains. The motility proteins SwmA and SwmB from *Synechococcus* sp. WH8102 (McCarren and Brahamsha, 2007) comprised 1.7% and 0.13%, respectively, of the exoproteome of this strain. The giant protein SwmB (10 791 amino acids in length) was previously visualized in the membrane fraction (McCarren and Brahamsha, 2007) but not resolved in Fig. 2B. This protein shared a low identity with the MS‐detected protein EAU73526 in *Synechococcus* sp. RS9916 (0.15% of the exoproteome), a much smaller protein of only 1159 amino acids in length but which contains a conserved flagellar‐like domain. Seven other giant proteins (> 2000 amino acids in length) were also detected during this study and are further discussed in the Appendix S1. The large protein, EAR17767.1 (1961 amino acids in length; 0.67% of the exoproteome), from *Synechococcus* sp. WH7805 contains up to three different peptidase domains, two cadherin adhesion repeats and five tryptophan‐rich repetitive domains, highlighting the complexity of these large proteins (Fig. 3C). Protein EAR19380.1, abundantly detected (2.2%) and unique to *Synechococcus* sp. WH7805, also contained a serralysin peptidase M10 domain, which commonly binds calcium in haemolysin‐like proteins (pfam08548).

#### Other functions

Alkaline phosphatase enzymes were only detected in *Synechococcus* sp. WH5701 and *Synechococcus* sp. WH8102. While *Synechococcus* sp. WH5701 contained only one alkaline phosphatase in its exoproteome (EAQ75607.1, third most detected protein, 1.9%), *Synechococcus* sp. WH8102 expressed three different potential isoenzymes (NP_898480.1, 1.8%; NP_898479.1, 0.4%; and NP_896291.1, 0.1%) and a phytase‐like protein (NP_896855.1, 0.3%).

#### Exported hypothetical proteins

This group of unknown proteins, representing on average 18% of the experimental exoproteomes across all eight strains analysed, but rising to almost 40% of the exoproteome of *Synechococcus* sp. RS9917 (Table 3), comprises proteins of a relatively small size (150–240 amino acids in length). Interestingly, we found abundantly exported hypothetical proteins that are present in the genomes of most marine picocyanobacteria (e.g. protein cluster 8 and 18 of Table S4) or that were specific to the genus *Synechococcus* (e.g. clusters 17 and 24 of Table S4), all being abundantly detected in most of the analysed exoproteomes. Other highly detected exported hypothetical proteins showed a higher strain‐specificity, e.g. clusters 19 (8.9% abundance) and 35 (4.7%) were specific to strains BL107 and RS9917 respectively.

We further verified the identity of the most abundant proteins in *Synechococcus* exoproteomes seen in Fig. 2B using chymotrypsin digestion. This highlighted the importance of the swimming proteins SwmA and SwmB in *Synechococcus* sp. WH8102, the chitinase‐like enzyme in *Synechococcus* sp. RS9917 and WH7805, and the haemolysin‐related proteins in *Synechococcus* sp. WH7805 (further details on this analysis can be found in the Appendix S1).

### Characterization of the *S* *ynechococcus* WH8102 exoproteome following growth under different environmental conditions

In addition to growth of (i) *Synechococcus* WH8102 over a 4 day period under the standard conditions mentioned above, exoproteomes were also analysed following (ii) co‐culture of WH8102 with the α‐proteobacterium *R. pomeroyi* DSS‐3, (iii) growth of WH8102 over a 10 h incubation period, (iv) 10 h dark incubation of WH8102 and (v) WH8102 cultures infected for 10 h with cyanophage S‐RSM4. Shotgun proteomic analysis of these five different conditions (each performed in triplicate) generated 24 914 MS/MS‐identified peptides. In this case, on average, 101 proteins per sample were validated (Table S5). Comparative proteomics between each of the relevant conditions was carried out (Table S6) and are commented upon hereafter.

#### Stand‐alone growth versus co‐culture growth

Co‐culture with *R*. *pomeroyi* revealed strong upregulation of four hypothetical proteins under co‐culture conditions: NP_896260.1 with no known function (8.4×); and NP_898392.1 (5.2×), NP_896974.1 (5.1×) and NP_898441.1 (4.3×), which contained domains related to virulent factor proteases and adhesion. These latter three proteins together with two haemolysin‐like proteins that were also upregulated in the presence of *R. pomeroyi* DSS‐3 (NP_898382.1, 3.3×; NP_898498.1, 1.8×) are indicative of a predator‐type response by *Synechococcus* sp. WH8102 to the presence of the heterotroph. The haemolysin‐like protein (NP_898382.1) also contains an alpha‐tubulin suppressor‐like domain, as do two other proteins exported under co‐culture conditions (NP_897657.1, 2.6×; NP_897658.1, 2.4×), which potentially hints at a role in restructuring the cell surface envelope in *Synechococcus* sp. WH8102. The motility proteins SwmA and SwmB were also upregulated (NP_896180.1, 1.7×; NP_897046.1, 3.0×) together with three alkaline phosphatases (NP_896291.1, 4.1×; NP_898479.1, 2.2×; NP_898480.1, 2.1×) and a phytase‐like protein (NP_896855.1, 3.2×), the latter proteins, at least, suggesting phosphate depletion as was seen previously when this strain was grown in the presence of *Vibrio parahaemolyticus* (Tai *et al*., 2009). Unlike the upregulated proteins, which were all potentially exported proteins, downregulated proteins found in the exoproteome were mostly cytoplasmic, i.e. lacked a specific signal sequence (43 out of 56, Table S6).

#### Standard (4 days) versus shorter incubation time (10 h)

As expected, a reduced number and lower concentration of leaked proteins (i.e. downregulation of numerous cytoplasmic proteins) occurred with shorter incubation time. Interestingly, two porins were strongly upregulated (NP_898316.1, 15.4×; NP_898315.1, 6.9×) together with active ABC transporters for iron and urea (9.8× and 2.9× respectively).

#### Standard versus dark conditions

Upregulated exoproteins under dark conditions were mostly predicted cytoplasmic proteins indicating an increased leakage of proteins to the milieu (36 out of 46, Table S6). The three abundantly detected alkaline phosphatases, a phytase‐like enzyme and the motility proteins SwmA and SwmB were strongly downregulated and almost undetectable under dark conditions. Noteworthy was the downregulation of all the interaction proteins abundantly observed when co‐cultured with the heterotroph (i.e. the three proteins containing alpha‐tubulin suppressor domains, haemolysins and metallopeptidases). This was visually confirmed by SDS‐PAGE and noted in other strains, i.e. *Synechococcus* sp. WH7805 where bands e and f of Fig. 3C containing type I secretion protein (EAR18050.1), haemolysin (EAR19380.1) and chitinase (EAR19694.1) disappeared.

#### Standard versus phage infection

A low number of exoproteins showed a variation in abundance following infection with cyanophage S‐RSM4. The motility proteins SwmA and SwmB showed a moderate downregulation (2.2× and 2.0× respectively), while the already abundantly detected substrate binding proteins of the phosphate ABC transporter NP_897906.1 and NP_897111.1 showed an increase (2.0× and 1.3× respectively).

### *S* *ynechococcus* co‐cultures with heterotrophs

**Figure 4:**
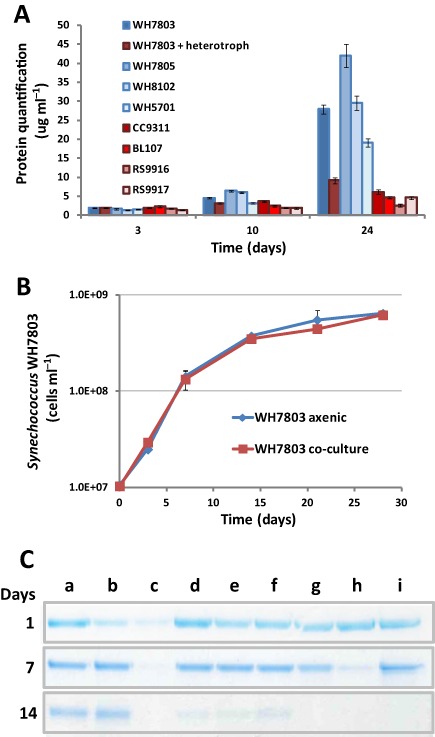
*S* *ynechococcus*‐heterotroph co‐culturing. (A) Quantification of proteins accumulated in the milieu of the eight different *S* *ynechoccus* strains used in this study. Bars are an average of three biological replicates. Red tones represent cultures where heterotrophs were present and blue tones represent axenic cultures. (B) Growth of axenic *S* *ynechococcus* sp. WH7803 under standard conditions and in co‐culture with *R* *. pomeroyi* DSS‐3. (C) BSA degradation in different culture conditions: (a) ASW control; (b) culture with *S* *ynechococcus* sp. WH7803; (c) co‐culture with *S* *ynechococcus* sp. WH7803 and *R* *. pomeroyi* DSS‐3; (d–i) *R*. *pomeroyi* DSS‐3 cultured in media: (d) ASW; (e) ASW supplemented with NH_4_ (9 mM); (f) ASW with yeast extract (0.005%); (g) ASW with yeast extract and pyruvate (0.5%); (h) 1/10 marine broth in ASW; (i) marine broth. BSA was used at a final concentration of 0.02% w/v). Cultures were filtered through a 0.22 μm pore size membrane and proteins from 20 μl of filtered milieu were resolved by 10% SDS‐PAGE.

All of our data indicated a progressive accumulation of cytoplasmic proteins in the exoproteomes of axenic cultures but not in non‐axenic *Synechococcus* cultures, i.e. the non‐exported proteins detected in the exoproteomes of *Synechococcus* sp. WH8102 increased from 3.3 ± 0.6% after 10 h incubations to 52.2 ± 0.8% after 4 day incubations, but for the latter this was reduced to 33.2 ± 2.4% when *R. pomeroyi* DSS‐3 was present in the culture. We monitored the accumulation of proteins in the exoproteomes of all eight analysed *Synechococcus* strains (Fig. 4A). As expected, elevated concentrations of proteins in the exoproteome were only observed in axenic *Synechococcus* cultures during the stationary phase of growth (between 19 and 42 μg ml^−1^ depending on strain). Interestingly, the concentration of exoproteins in axenic *Synechococcus* sp. WH7803 supernatants was reduced threefold when grown with *R. pomeroyi* DSS‐3 (27.9 versus 9.2 μg ml^−1^), the latter concentration comparable with that seen in other non‐axenic *Synechococcus* cultures (Fig. 4A). Growth of *Synechococcus* sp. WH7803 in axenic versus *R. pomeroyi* DSS‐3 co‐culture (Fig. 4B) was very similar when monitored by cell counting, with doubling times of 38.2 and 40.1 h, respectively, and comparable cell yields after 28 days of incubation (6.4 × 10^8^ and 6.2 × 10^8^ cells l^−1^ respectively). These results indicate that the higher number of predicted cytoplasmic proteins in the exoproteomes and the higher concentration of protein accumulated in stationary phase is not due to an increase in cell lysis of axenic cultures, but to a faster turnover of these proteins when heterotrophic bacteria are present.

Finally, we analysed the proteolytic activity of *Synechococcus* sp. WH7803 and *R. pomeroyi* DSS‐3 by monitoring the degradation of BSA added to the culture. The results reported in Fig. 4C show that *Synechococcus* sp. WH7803 was unable to hydrolyse BSA after 14 days incubation, whereas *R. pomeroyi* DSS‐3 showed almost complete degradation of the protein. Most interestingly, the fastest degradation of BSA was seen in *R. pomeroyi* DSS‐3‐*Synechococcus* sp. WH7803 co‐cultures (Fig. 4C, lane c).

## Discussion

Exoproteomes are good indicators of microbial lifestyle strategies. Here, we carried out a thorough theoretical and experimental analysis of marine picocyanobacterial exoproteomes. The predicted exported fraction of these key photosynthetic primary producers (40% of their encoded proteins) highlighted two major points: (i) the current lack of functional knowledge regarding exported proteins (54% of the theoretical exported CDS are annotated as ‘hypothetical proteins’ whereas these unknown proteins only represented 35% of the cytosolic fraction) and (ii) this exported fraction appears to be a major factor in dictating the genomic and functional distinctness of these closely related organisms (only 57% of the exported CDS contain a homologue in other *Synechococcus* stains versus 73% in the cytosolic fraction). Exposure to the outside world determines their strategy for interaction, and ultimately accomplishes niche partitioning of closely related organisms not only to avoid direct competition, but also to evade grazing or phage infection. For example, diversification of potentially membrane‐exposed proteins, which might act as phage receptors, across closely related strains would avoid catastrophic lysing of members of the entire genus at any given time and location. In this study, we used model strains isolated at different times and locations, but recently published data showing the distinctness in coexisting *Prochlorococcus* subpopulations (Kashtan *et al*., 2014) shows how this may actually be relevant in natural environments.

Experimental analysis of eight marine *Synechococcus* strains confirmed how each strain has a distinct adaptation through the exported protein fraction. For example, we were surprised that the pili‐like structure encoded by four of the strains was only detected in *Synechococcus* sp. WH7803 and represented as much as 19% of this strains exoproteome. Exported proteins involved in microbial interactions showed the highest diversification, with many of the genes encoding these proteins unique to specific strains. Other previously published examples include small ribosomally synthesized peptides with toxic effects (Paz‐Yepes *et al*., 2013; Zhang *et al*., 2014) through to giant proteins involved in motility and grazing evasion (McCarren and Brahamsha, 2007; Strom *et al*., 2012). Giant proteins were abundantly detected in our MS/MS analysis although became less relevant after normalizing the data to their molecular size. These giant proteins (up to four different ones detected in *Synechococcus* sp. RS9916) were classified as interaction proteins because of the adhesion and haemolysin‐like domains they contain. This diverse set of peculiar large proteins, that rarely share similarities among strains, are thought to play a role in shielding cells from potential threats (Scanlan *et al*., 2009).

Evolution of intracellular proteins is restrained because of protein crowding and numerous protein–protein interactions in the cell that may well not occur in exported proteins, and consequently their evolution is more prone to be highly divergent. Furthermore, extracellular enzymes with a simple genetic structure are known to be rapidly gained and lost through evolution (Zimmerman *et al*., 2013), facilitating the acquisition of functional distinctness via this extracellular fraction. Horizontal gene transfer of large genetic islands may facilitate the acquisition of these simple functions, but also of more complex operons or genes encoding for giant proteins such as those detected here (Dufresne *et al*., 2008). In this respect, the abundant detection of a chitinase‐like endo‐1,4‐beta‐glucanase (in the exoproteomes of *Synechococcus* spp. RS9916, RS9917, WH7803 and WH7805) and potential exoproteases (e.g. EAR17767.1 produced by *Synechococcus* sp. WH7805) may have been acquired and conserved as a result of a beneficial selection. It is interesting that microorganisms such as marine *Synechococcus*, with a known preference for inorganic nutrients, produce exo‐chitinases and proteases that, ultimately, may have a role in nutrient supplementation (i.e. as additional nitrogen sources) as part of a mixotrophic lifestyle. Indeed, picocyanobacteria are known for their ability to acquire amino acids and carbohydrates (Montesinos *et al*., 1997; Zubkov *et al*., 2003; Mary *et al*., 2008; Muñoz‐Marín *et al*., 2013) at low nanomolar concentrations, potential products of chitinase or protease activity. It is also possible that these exoenzymes may have a role in eliminating competitors, especially diatoms, because the latter contain chitin in their silica cell wall (Brunner *et al*., 2009). Type IV pilins, like the one detected in *Synechococcus* sp. WH7803, have also been reported to have a potential role in chitin adhesion in *Vibrio* (Frischkorn *et al*., 2013). Hence, picocyanobacteria may appear to be more hostile microorganisms than previously anticipated.

The acquisition of inorganic nutrients is a key process carried out at the cellular membrane of marine cyanobacteria. Proteins involved in transport are commonly detected in exoproteome studies (Christie‐Oleza and Armengaud, 2010; Johnson‐Rollings *et al*., 2014), and, therefore, it was not surprising to find that substrate binding proteins of ABC transporters for iron and phosphate were among the most abundant proteins detected over the entire study. The fact that different isoforms of the phosphate binding protein component of the ABC transporter and several alkaline phosphatases co‐occurred in some of the *Synechococcus* exoproteomes suggests the experimental cultures were actively scavenging P from their environment, likely both for growth and storage (Moore *et al*., 2005; Mazard *et al*., 2012).

Overall, our experimental data indicate that non‐exported proteins of *Synechococcus* can be found in the extracellular milieu. Whether the observed leakage is via cell lysis, unknown cell loss mechanisms or by the large quantity of vesicles these organisms are known to release (Biller *et al*., 2014) is unclear. Whatever the mechanism, axenic cultures accumulate larger amounts of cytoplasmic and thylakoid‐like proteins in their milieu than non‐axenic cultures. This could be due to a lower incidence of *Synechococcus* cell lysis when co‐cultured with a heterotroph, or that the heterotrophic bacteria present in co‐cultures are capable of degrading the more labile cytoplasmic proteins and, hence, these have a faster turnover in the milieu. Despite the difficulty to experimentally ascertain which is the most likely scenario, the identical growth rate seen in axenic and co‐cultured *Synechococcus* indicates similar cell lysis rates. Interestingly, the heterotroph *R. pomeroyi* DSS‐3 showed high exoprotease activity when grown in co‐culture with *Synechococcus*, indicating increased potential for removing proteins from the milieu. Picocyanobacterial cell debris and vesicles are known to support heterotrophic growth (Biller *et al*., 2014), perhaps via some type of mutualistic interaction. The presence of a heterotroph not only removed leaked cytoplasmic proteins from the exoproteome, but in *Synechococcus* sp. WH8102 also caused the upregulation of a large set of interaction proteins (i.e. swimming proteins, virulent factor‐like proteases, haemolysin and adhesion proteins). The other condition tested which showed a strong influence on the exoproteome was dark incubations. Probably pushed by a decrease in its energy potential, *Synechococcus* drastically reduced protein export, especially those with roles in interaction (i.e. involved in swimming, haemolysin production, alkaline phosphatase, see Fig. 2C). This form of dormancy could be a strategy not only to save energy, but may also disguise cells at night by reducing the prevalence of viral receptors and grazer recognition proteins, being a possible explanation as to why some cyanophage are unable to absorb their host in the dark.

Our experimental exoproteomes draw some parallels to those observed in the natural environment from a metaproteome of high molecular weight dissolved organic matter in surface seawaters of the South China Sea (Dong *et al*., 2013). Despite the fact that only 17 of 367 identified polypeptides could be confidently assigned to cyanobacteria, eight were of unknown function, two were nitrogen membrane transporters, and four were directly involved in photosynthesis and carbon fixation.

In conclusion, the exoproteomes of *Synechococcus* highlight several particularly interesting ecological traits of marine picocyanobacteria: (i) the exported fraction gives a specific functional distinctness to these phototrophs for potential niche partitioning and diversifying receptors to evade prey, (ii) the large number of uncharacterized proteins these organisms have to interact with their environment, (iii) the potential hostile repertoire of exoenzymes for eliminating direct competitors or to supplement nutritional needs through mixotrophy, (iv) their ability to strongly modify their exoproteome under different conditions, i.e. light/dark or growth with heterotrophs, and (v) how the exoproteomes of these microorganisms can support the marine food web.

## Experimental procedures

### Bacterial strains and growth conditions

Marine *Synechococcus* strains BL107 (clade IV), CC9311 (clade I), RS9916 (clade IX), WH5701 (subcluster 5.2), WH7803 (clade V), WH7805 (clade VI) and WH8102 (clade III) were routinely grown in ASW medium (Wilson *et al*., 1996) at 22°C with a light intensity of 10 μmol photons m^−2^ s^−1^. *Synechococcus* sp. RS9917 was grown in ASW medium supplemented with 5 mM (NH_4_)_2_SO_4_ because this strain cannot utilize nitrate for growth (Fuller *et al*., 2003). *Synechococcus* spp. WH5701, WH7803, WH7805 and WH8102 are all axenic strains while the remaining strains are clonal but non‐axenic. *Synechococcus* sp. WH8102 was also grown (i) in co‐culture with the heterotrophic bacterium *R. pomeroyi* DSS‐3 (the latter at a density of 10^6^ cells ml^−1^), (ii) in the dark for 10 h and (iii) in the presence of cyanophage S‐RSM4 (at a multiplicity of infection of 0.25) for 10 h. In this latter case, exoproteomes were prepared prior to cell lysis. Co‐cultures of *Synechococcus* sp. WH7803 with *R. pomeroyi* DSS‐3 were performed with similar starting cell concentrations. *Synechococcus* sp. WH7805 was similarly incubated in the dark.

### Preparation of exoproteomes for nanoLC‐MS/MS analysis

Cultures for exoproteome analysis were prepared by spinning the cells (3000 g at room temperature during 15 min) and gently re‐suspending them in fresh media at a final concentration of 3 × 10^7^ cell ml^−1^. Cultures were then left to grow in standard conditions to cell densities of 10^8^ cells ml^−1^. At this point, cultures were subjected to centrifugation at 3000 g for 15 min at room temperature. Supernatants were carefully removed and then gently filtered through 0.22 μm pore size filter units (Sterivex‐GV, Millipore) to eliminate any remaining cells. Proteins in the remaining milieu were concentrated and purified by precipitation with trichloroacetic acid and run on SDS‐PAGE as previously described (Christie‐Oleza and Armengaud, 2010). Trypsin in‐gel proteolysis of the entire exoproteome was performed for the shotgun proteomics analysis as recommended (Hartmann *et al*., 2014). NanoLC‐MS/MS experiments were performed using a LTQ‐Orbitrap XL hybrid mass spectrometer (ThermoFisher) coupled to an UltiMate 3000 LC system (Dionex‐LC Packings). Conditions used were those previously described (de Groot *et al*., 2009).

### MS/MS database search, abundance and comparative analysis

Compiled MS/MS spectra were searched against the annotated coding domain sequences of each strain downloaded from the NCBI (10/07/2012). Searches were carried out with MASCOT 2.2.04 software (Matrix Science) using parameters previously established (Christie‐Oleza *et al*., 2012). MASCOT results were parsed and peptides were filtered at a *P*‐value below 0.05. A protein was considered validated when at least two unique peptides were detected in the same experiment. A false‐positive rate below 0.1% for protein identification was estimated using a reverse decoy database as previously done (Christie‐Oleza *et al*., 2012). Protein quantification by spectral abundance was done as previously described (Liu *et al*., 2004). For normalized spectral abundance factors of each protein, spectral counts assigned to each polypeptide were divided by its molecular weight. Values were then normalized by the total sum corresponding to all the polypeptides detected with two or more non‐redundant peptides. Statistical comparisons between *Synechococcus* sp. WH8102 mass spectrometry‐detected exoproteins following growth under different conditions was carried out with the TFold method of the PatternLab program (Carvalho *et al*., 2012). A BH‐FDR statistical test was calculated to evaluate the global false discovery rate for each comparison.

### Protein quantification and BSA‐degradation experiments

Protein was quantified with QuantiPro BCA Assay kit (Sigma‐Aldrich). Degradation of Bovine serum albumin BSA was used to determine the potential exoprotease activity of cultures. Experiments were performed in 48‐well plates with 800 μl culture (*Synechococcus* sp. WH7803 at cells ml^−1^ and *R. pomeroyi* DSS‐3 at 10^7^ cells ml^−1^) and 200 μl 0.1% (w/v) BSA in ASW medium. ASW medium alone was used as a control. After culture incubation in standard conditions (see above), a 20 μl volume of supernatant was resolved and visualized by SDS‐PAGE as described in Christie‐Oleza and Armengaud (2010).

### Protein *in silico* analysis

The theoretical exoproteome of the eight *Synechococcus* strains used in the experimental study plus four *Prochlorococcus* strains (MED4, MIT9303, MIT9312 and MIT9313) and SAR11 (*Pelagibacter ubique* HTCC1062) was determined based on three prediction tools: (i) the SignalP 4.0 server for predicting N‐terminal signal peptides for secretion (Petersen *et al*., 2011), (ii) the SecretomeP 2.0 server for predicting proteins exported by non‐classical systems (Bendtsen *et al*., 2005) and (iii) the LipoP server for predicting lipoproteins (Juncker *et al*., 2003). PSORTb 3.0 was used to determine subcellular location of the proteins (Yu *et al*., 2010). Local BLASTp analyses were done with the BioEdit BLAST tool v.7.0.5.3 (Hall, 1999) using default parameters and an E‐value cut‐off < 10^−20^. Conserved protein domains and motifs were determined using the Conserved Domains tool at the NCBI (http://www.ncbi.nlm.nih.gov/Structure/cdd/wrpsb.cgi).

## Supporting information


**Table S1.** Exported proteome prediction of eight *Synechococcus* strains, four *Prochlorococcus* strains and SAR11.

**Table S2.** Homology of each CDS with the closest CDS in the other 11 strains (plus SAR11) with tentative functions. The subset of giant proteins can be found in Table S2B.

**Table S3.** MS‐detected peptides from the exoproteomes of eight *Synechococcus* strains and list of polypeptides detected with two or more peptides.

**Table S4.** LC‐MS/MS detected proteins from the exoproteome of eight different *Synechococcus* strains grouped by abundance and homology.

**Table S5.** MS‐detected peptides from the exoproteome of *Synechococcus* WH8102 under different conditions and list of polypeptides detected with two or more peptides.

**Table S6.** Comparative proteomics of the exoproteomes of *Synechococcus* WH8102 submitted to different conditions.

**Appendix S1.** Supplementary data.
