# Supplementary material for: Functional distinctness in the exoproteomes of marine S ynechococcus
Source: Environ Microbiol. 2015 Apr 15;17(10):3781–94. doi: 10.1111/1462-2920.12822 (PMC4949707; doi:10.1111/1462-2920.12822)
Supplement: Supplementary file 7 — Appendix S1. Supplementary data. [file EMI-17-3781-s007.docx]

**Supplementary Data for**

**Functional distinctness in the exoproteomes of marine *Synechococcus***

Joseph A. Christie-Oleza^1*^, Jean Armengaud^2^, Philippe Guerin^2^, David J. Scanlan^1^

^1^ School of Life Sciences, University of Warwick, Coventry CV4 7AL, UK

^2^ CEA, DSV, IBiTec-S, SPI, Li2D, Laboratory "technological Innovations for Detection and Diagnostic", Bagnols-sur-Cèze, F-30207, France

^*^ Corresponding author: j.christie-oleza@warwick.ac.uk

**Analysis of the theoretical exoproteome**

The theoretical exoproteome for the twelve picocyanobacteria was grouped in eight functional protein clusters (Table 2 and S2). In terms of functional grouping, a major part of the theoretical exported fraction is made of proteins of unknown function (though the 54% figure above reduced to 51.2% after finding homologues in other strains see Table S2). Most components of the photosynthetic or electron transport chain for energy generation are linked to the membrane, and hence are included in the exported fraction (9.6% of the exported fraction, Table 2). Some proteins involved in dealing with oxidative stress were also predicted to have a transmembrane component or to be exported to the periplasm. Transport-related systems represent an important part of the theoretical exported fraction (11.8%, Table S2), being mostly transporters for acquiring inorganic nutrients (e.g. nitrogen, phosphorus and trace metals). Transporters for obtaining organic molecules (e.g. carbohydrates and amino acids) are also commonly found in all strains except for the smaller-sized *Prochlorococcus* genomes MED4 and MIT9312 (see Table S2) as previously noted in Scanlan *et al.*, (2009). Interestingly, despite their streamlined genomes, these twelve picocyanobacteria still encode proteins involved in cell-to-cell interactions (i.e. pili or fimbriae), RTX-like proteins (Linhartova *et al*., 2009) and ‘giant’ exported proteins (Table S2b) with generally poorly understood functions (Reva & Tummler, 2009; Scanlan *et al.*, 2009).

**Giant proteins**

Encoded in the genomes and theoretically exported: The exported RTX-like proteins present in marine bacteria are usually large polypeptides (some of over 2,000 amino acids) and have the characteristic glycine/aspartic acid-rich nanopeptide repeat that binds Ca^2+^ together with adhesion- or metalloprotease-like domains. Leaving modular polyketide synthase proteins aside, other giant proteins (>2,000) are always predicted to be exported, usually autotransported through the membrane. Despite the burden for synthesising these enormous polypeptides or just conserving their large genes, they are present in one to six copies in seven of the eight *Synechococcus* strains (not observed in WH5701, the least similar of the *Synechococcus* genera) (Table S2b). Strain RS9916 encodes six of these giant exported proteins, three of them being over 7,000 amino acids long. Strikingly, *Synechococcus* sp. RS9917 encodes a protein 28,178 amino acids in length (ZP_01080684.1). On the other hand, only one giant protein (2082 aa in strain MIT9303) was found amongst the four *Prochlorococcus* strains. The streamlined genome of SAR11 also contains a protein 7,317 amino acids in length. The function of these giant proteins is not clear but they are thought to have a role in conferring protection to the cell by shielding it from potential threats or via adhesion. The only characterised giant protein within these strains, that of SwmB in *Synechococcus* sp. WH8102 (NP_897046.1), appears to be involved in swimming motility and avoiding predation by grazers (McCarren & Brahamsha, 2007; Strom *et al.*, 2012).

Giant exported proteins experimentally detected by LC-MS/MS: A total of eight giant proteins (>2,000 amino acids in length) were detected in our proteomic survey. The giant protein SwmB (10,791 amino acids in length) shared a low identity with the MS-detected protein EAU73526 in *Synechococcus* sp. RS9916 (0.15% of the exoproteome), a much smaller protein of only 1,159 amino acids in length but which contains a conserved flagellar-like domain. Other giant proteins mostly contained an autotransporter domain at the C-terminal end of the protein and with a putative adhesion function. Thus, *Synechococcus* sp. RS9916 expressed four giant proteins in the exoproteome: EAU75567.1 (7,079 amino acids in length; 1.05% of the exoproteome), EAU73485.1 (4,603 amino acids in length; 0.08% of the exoproteome), EAU75184.1 (7,750 amino acids in length; 0.07% of the exoproteome) and EAU73487.1 (5,574 amino acids in length; 0.02% of the exoproteome). Protein EAU75567.1 showed some sequence identity with two other detected giant proteins in the exoproteomes of strains RS9917 (ZP_01078944.1; 9,144 amino acids in length; 0.32% of the exoproteome; 53% amino acid sequence identity) and WH7805 (EAR17372.1; 8,129 amino acids in length; 0.01% of the exoproteome; 23% identity).

**Verification of the identity of the most abundant proteins in *Synechococcus* exoproteomes**

Resolved protein bands by SDS-PAGE (labeled in Figure 2B) were digested with chymotrypsin (Roche) and the resulting peptides were identified by tandem mass spectrometry in order to verify the most abundant proteins. Exported proteins can sometimes be recalcitrant to classical proteomic identification protocol via trypsin digestion (Durighello *et al.*, 2014) and, hence, be negatively biased in shotgun-proteomic approaches. Seven resolved bands (Figure 2B) were cut and digested with chymotrypsin, a protease with orthogonal specificities compared to trypsin. The proteins were identified as i) *Synechococcus* sp. WH8102: band a, SwmA NP_896180.1; band b, was a mix of both SwmA NP_896180.1 and SwmB NP_897046.1; band c, phosphate ABC transporter NP_897111.1; ii) *Synechococcus* sp. RS9917: band d, chitinase ZP_01081204.1; iii) *Synechococcus* sp. WH7805: band e, type I secretion protein EAR18050.1; band f, was a mix of hemolysin EAR19380.1 and chitinase EAR19694.1; and iv) *Synechococcus* sp. WH5701: band g, alkaline phosphatase EAQ75607.1. All proteins identified following this approach corresponded to highly abundant proteins detected in our shotgun strategy although the swimming protein SwmA (NP_896180.1) with 1.7% abundance in our survey (band a in Figure 2B) and the hemolysin-like protein (EAR19380.1 with 2.2% abundance, band f) could have been slightly underestimated. Trypsin works ideally for proteomics as it generates peptides with length and ionizability characters perfectly compatible for tandem mass spectrometry. The average peptide size generated by tryptic digestions for all CDS in the eight *Synechococcus* strains was 10.4 amino acids in length. Nevertheless, most giant exoproteins and some interaction-like proteins (i.e. RTX-like, adhesion, exoprotease) were usually more recalcitrant to trypsin digestion as average peptide sizes ranged from over 20 to 75 amino acids in length. In this respect, trypsin digestion of the hemolysin EAR19380.1 of *Synechococcus* sp. WH7805 generated, on average, 57 amino acid-long peptides, whilst for SwmA and SwmB from *Synechococcus* sp. WH8102 peptides of 20 and 25 amino acids were generated, respectively.

BIBLIOGRAPHY

Durighello, E., Christie-Oleza, J.A., Armengaud, J. (2014). Assessing the exoproteome of marine bacteria, lesson from a RTX-toxin abundantly secreted by *Phaeobacter* strain DSM 17395. *PloS One* **9**: e89691.

Linhartova, I., Bumba, L., Masin, J., Basler, M., Osicka, R., Kamanova, J., *et al*. (2010). RTX proteins: a highly diverse family secreted by a common mechanism. *FEMS Microbiol Rev* **34**: 1076-1112.

McCarren, J., Brahamsha, B. (2007). SwmB, a 1.12-megadalton protein that is required for nonflagellar swimming motility in *Synechococcus*. *J Bacteriol* **189**: 1158-1162.

Reva, O., Tummler, B. (2008). Think big - giant genes in bacteria. *Environ Microbiol* **10**: 768-777.

Scanlan, D.J., Ostrowski, M., Mazard, S., Dufresne, A., Garczarek, L., Hess, W.R., *et al*. (2009). Ecological genomics of marine picocyanobacteria. *Microbiol Mol Biol Rev* **73**: 249-299.

Strom SL, Brahamsha B, Fredrickson KA, Apple JK, Rodriguez AG (2012). A giant cell surface protein in *Synechococcus* WH8102 inhibits feeding by a dinoflagellate predator. Environ Microbiol 14: 807-816.
